# Review of additive manufactured tissue engineering scaffolds: relationship between geometry and performance

**DOI:** 10.1186/s41038-018-0121-4

**Published:** 2018-07-03

**Authors:** Andrew Gleadall, Dafydd Visscher, Jing Yang, Daniel Thomas, Joel Segal

**Affiliations:** 1 Wolfson School of Mechanical and Manufacturing Engineering, Loughborough University, Loughborough, Leicestershire LE11 3TU UK; 2 Department of Plastic, Reconstructive and Hand Surgery, VU University Medical Center, Amsterdam Movement Sciences, Amsterdam, The Netherlands; 3 Faculty of Science, School of Pharmacy, University of Nottingham, University Park, Nottingham, NG7 2RD UK; 4 3Dynamic Systems, Heol Ty Gwyn Industrial Estate, Bridgend, CF34 0BQ UK; 5 Advanced Manufacturing Technology Research Group, Faculty of Engineering, University of Nottingham, University Park, Nottingham, NG7 2RD UK

**Keywords:** Fused deposition modelling, Bioprinting, 3D printing, Scaffold architecture, Tissue engineering constructs, Regenerative medicine

## Abstract

Material extrusion additive manufacturing has rapidly grown in use for tissue engineering research since its adoption in the year 2000. It has enabled researchers to produce scaffolds with intricate porous geometries that were not feasible with traditional manufacturing processes. Researchers can control the structural geometry through a wide range of customisable printing parameters and design choices including material, print path, temperature, and many other process parameters. Currently, the impact of these choices is not fully understood. This review focuses on how the position and orientation of extruded filaments, which sometimes referred to as the print path, lay-down pattern, or simply “scaffold design”, affect scaffold properties and biological performance. By analysing trends across multiple studies, new understanding was developed on how filament position affects mechanical properties. Biological performance was also found to be affected by filament position, but a lack of consensus between studies indicates a need for further research and understanding. In most research studies, scaffold design was dictated by capabilities of additive manufacturing software rather than free-form design of structural geometry optimised for biological requirements. There is scope for much greater application of engineering innovation to additive manufacture novel geometries. To achieve this, better understanding of biological requirements is needed to enable the effective specification of ideal scaffold geometries.

## Background

Worldwide, approximately 230 million major surgical procedures are performed on a daily basis [1]. Most of these procedures involve reconstruction, repair, or replacement of one or more damaged tissues or organs. Tissue engineering in combination with additive manufacturing has emerged as an alternative technique to regenerate damaged tissues and organs by developing patient-specific substitutes that restore, improve, or maintain tissue function [2]. Engineering of functional tissues or organs requires a scaffold, which acts as a template for tissue regeneration. Additive manufacturing can help build such a template in a layer-by-layer fashion and is particularly appropriate for reconstructive surgery for facial trauma because scaffolds with patient-specific anatomical geometries can be fabricated. Currently, materials that facilitate bone ingrowth are widely used in reconstructive surgery for the treatment of trauma, but tissue engineering scaffolds will see more widespread clinical usage as research and clinical translation continues.

One of the most common additive manufacturing technologies for tissue engineering scaffolds is material extrusion additive manufacturing, in which a variety of materials can be extruded including polymers, hydrogels, and ceramic pastes. For thermoplastics, the process is also known as fused deposition modelling (FDM). During material extrusion additive manufacturing, filaments are extruded from a nozzle and positioned relative to one another according to a pattern chosen by the user. Complex geometries and porous structures can be achieved with a fully interconnected network of pores, which is not possible with conventional fabrication techniques such as injection moulding or machining. Porosity is especially important in scaffold design because it allows blood vessel ingrowth, nutrient diffusion, oxygen transport, and waste removal, which are important factors for the regeneration of fully functional tissues [3].

In 2015, Gariboldi and Best [4] introduced four levels of geometry for tissue engineering scaffolds: (1) surface topography, (2) pore size and geometry, (3) porous networks, and (4) macroscopic pore arrangement, including the potential for functionally gradient structural geometry. Whilst the first level relates to surface features of 10 μm more or less, and is typically dictated by material choice and processing method, the other three levels are all primarily dependent on the scaffold geometry, which is dictated by the position and orientation of filaments. Here, the term “position and orientation of filaments” refers to the physical geometric placement of filaments within a scaffold relative to one another, for example, the angular orientation of filaments on different layers or the spacing between filaments. These aspects of scaffold geometry can be varied independently of the design of the overall external scaffold geometry.

Hutmacher first reported the use of additive manufacturing for tissue engineering scaffolds in 2000 [5]. His group produced scaffold designs with several filament orientations. Since then, extensive research has investigated different designs for scaffold geometry and the effect on tissue formation. Many features of scaffold geometry can be controlled by the position and orientation of filaments, including pore volume fraction [6, 7], pore size [5, 7], pore shape [5, 8], mechanical properties [9, 10], and functional gradients of these properties [7, 11]. These are all important factors for tissue engineering scaffolds. Importantly, several studies have shown that scaffold geometry affects biological responses including cell seeding [7], cell proliferation [9, 12], and tissue formation [13].

This review identifies how the position and orientation of filaments, which also referred to in the literature as print path, lay-down pattern, or “scaffold design”, affect the porosity, mechanical properties, and biological performance of tissue engineering scaffolds produced by material extrusion additive manufacturing. First, an overview of material extrusion additive manufacturing technology and software is given. Next, the effect of scaffold geometry on porosity, mechanical properties, and biological performance is reviewed. Finally, innovative strategies for polymer deposition are discussed, and a future outlook is given.

## Material extrusion additive manufacturing

**Fig. 1:**
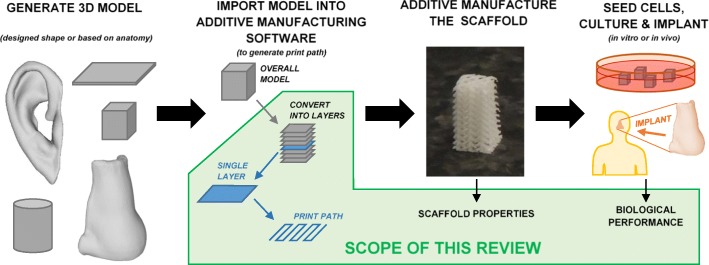
Overall procedure for additive manufacturing a tissue engineering construct. The scope of this review is to consider how the print path (equivalently referred to as filament orientation, lay-down pattern, or “scaffold design”) affects scaffold properties and performance

The overall process of additive manufacturing a tissue engineering scaffold is shown in Fig. 1. A three dimensional (3D) model is generated according to medical images or an engineering design. This model is imported into additive manufacturing software, which controls the overall deposition strategy used to produce the scaffold (print path, temperatures, nozzle travel speed, etc.). The scaffold is then manufactured and typically seeded with cells before either being implanted into the body or cultured in vitro for cell proliferation and tissue formation. This section gives an overview of commonly used material extrusion additive manufacturing technologies, materials, and software.

### Additive manufacturing technologies and materials

Many different manufacturing processes are used to fabricate scaffolds for tissue engineering. For simple geometries (e.g. sheet form), casting may be utilised with particle leaching or phase separation to achieve porosity, or processes related to woven/non-woven fabrics such as electrospinning may also be utilised [14]. For the reproduction of patient-specific anatomical geometries, however, the free-form nature of additive manufacturing processes makes them more appropriate. A wide range of additive manufacturing processes have been reviewed elsewhere [15, 16]. The present review focuses on material extrusion additive manufacturing, in which material is extruded through a nozzle.

A wide range of materials, with a vast range of properties, have been used for additive manufacturing tissue engineering scaffolds including metals, ceramics, polymers, natural materials, and composites. Other reviews have focused on the properties of different scaffold materials [2, 15], including the cell response to different materials and other important factors such as culture conditions and growth factors [17, 18]. The mechanical properties of different materials used in scaffolds produced by material extrusion additive manufactured are discussed in relation to biological tissues in the “Effect of scaffold geometry on mechanical properties” section.

### Material extrusion additive manufacturing technologies

**Fig. 2:**
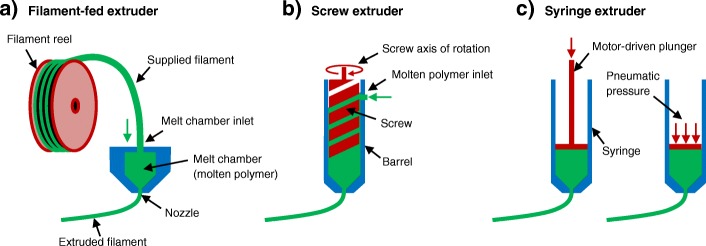
Three different types of extruders are illustrated schematically. **a** Filament-fed extruder. **b** Screw extruder. **c** Syringe extruders with either a mechanically driven plunger or pneumatic pressure plunger

Currently, there are three main material extrusion additive manufacturing technologies used to print scaffolds for tissue engineering as shown in Fig. 2: (1) filament-fed extruders, (2) screw extruders (typically fed by melted polymer pellets), and (3) syringe extruders (typically filled with a hydrogel or polymer pellets). Most commercial bioprinters use several different combinations of these extrusion technologies. They are capable of printing living cells in combination with various biomaterials for tissue fabrication.

#### Filament-fed extruders

Filament-fed additive manufacturing systems use reels of polymer filaments that typically have a diameter of 1.75 or 2.85 mm. These filaments are fed into a heated melting chamber which is attached to the nozzle. The rate of material extrusion from the nozzle is directly dictated by the rate at which filament is fed off the reels into the melting chamber, due to fundamental principles of volume conservation. Additive manufacturing software controls the extrusion rate based on the desired diameter of extruded filaments and the speed at which the nozzle is moving. This is the most common extrusion method for material extrusion additive manufacturing due to the low hardware costs and capability for a wide range of polymer melting temperatures. A major drawback of a filament-fed approach is that material options are limited to those that can be purchased in filament form.

#### Screw extruders

In screw extrusion additive manufacturing systems, polymer granules are fed into a screw that is surrounded by a close-fitting sleeve, called a barrel. As the screw rotates, molten polymer is forced through the nozzle at the end of the barrel. The rate of material extrusion from the nozzle is dictated by the screw rotation speed. The increased complexity of hardware for this extrusion method results in greater costs versus filament-fed printers. In addition to polymer granules, screw extruders can accommodate materials in paste form although this is less common.

#### Syringe extruders

In syringe extrusion additive manufacturing systems, the material is placed into a syringe and the printer depresses the plunger at a controlled rate to extrude filaments. Syringes are typically filled with a viscous material such as a hydrogel. Thermosoftening polymer granules may be used if a heated jacket heats the syringe to melt the polymer granules in situ before printing. Polycaprolactone has a low melting point (≈ 60 °C) so is ideal for typical syringes, although other polymers with higher melting points (e.g. > 200 °C) are feasible with stainless steel or glass syringes.

There are two main types of syringe extrusion systems: those which apply pneumatic pressure to the plunger and those which depress it by mechanical displacement with an electric motor. A key difference between these two methods is that mechanical displacement allows for more direct control over the volumetric extrusion rate (mm^3^/s) whereas in pneumatic printers, the extrusion rate depends on a complex interplay between needle geometry, material viscosity, pneumatic pressure, and obstruction by previously extruded filaments. In relation to the scope of this review, theoretically, more controlled filament geometries and scaffold structures may be achieved with mechanical plunger displacement due to the more direct control over extrusion rate. The benefit of pneumatic systems is that the forces applied to the material are known, which is important when considering shear stress in cell-laden hydrogels.

#### Multiple extruders

Bioprinters typically include several different extruders in one machine so that multiple materials can be printed during a single fabrication process. This is useful for co-printing scaffolds with cell-laden hydrogels and to create tissues that consist of multiple cell types and materials. Multiple materials may also be used to achieve gradient structures with a gradual transition from one material to another.

### Additive manufacturing software

Each additive manufactured system is supplied with its own software, and the capabilities vary greatly among different manufacturers. Traditional filament-fed systems for general engineering applications are typically supplied with software focused on achieving a high-quality surface finish for a wide range of part geometries. These softwares provide numerous options to control the internal structure, referred to as the infill, which consists of sparse filaments that provide mechanical integrity whilst minimising polymer usage, printing time, and part weight. However, they do not generally allow direct control over individual filaments. Software supplied with bioprinters is more focused on controlling the microscale geometry of scaffold pores and may enable precise positioning and material selection for each individual filament by allowing the user to manually draw the print path electronically within the software (e.g. BioCAD, regenHU Villaz-St-Pierre, Switzerland) [19]. Due to the needs of clinical applications, bioprinter softwares are also suitable for complex anatomic geometries [20].

For many material extrusion additive manufacturing systems, third-party software such as Slic3r [21] or CAD/CAM packages such as PrimCAM [22] can be used, as demonstrated by Schuurman et al. [23] for a polymeric scaffold with multiple hydrogels in a single construct. However, third-party software can only be used if the machine control code (GCODE) is not of a proprietary format and if the system allows custom GCODE to be uploaded to the machine.

**Fig. 3:**
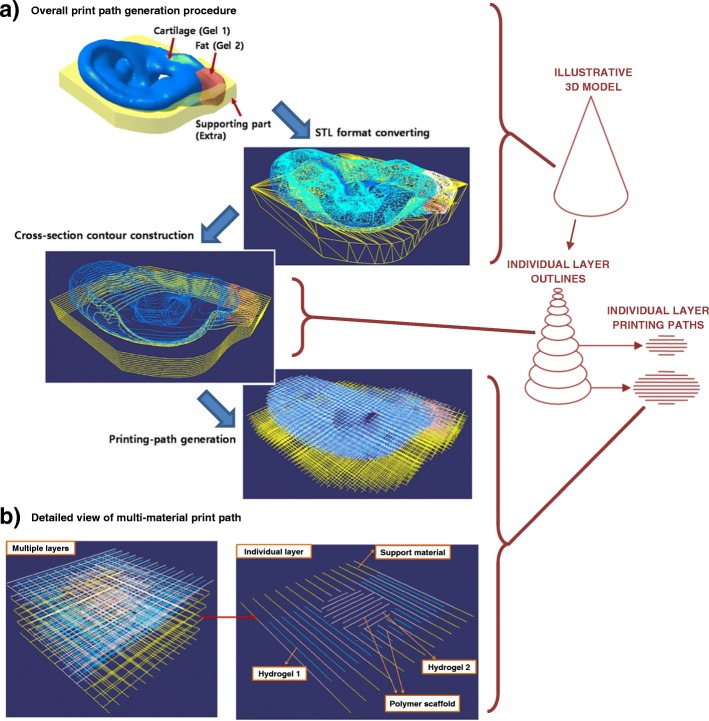
A custom print path generation algorithm was developed by Jung et al. [24]. It was demonstrated for **a**) an ear geometry and **b**) a simplified multi-material construct, in which four different materials were printed on each individual layer. Colours of the filaments indicate polymer scaffold (white), two different cell-laden hydrogels (blue and red), and the polymer support material (yellow), which supports overhangs on subsequent layers. An illustrative 3D model for a cone is shown on the very right of the figure to illustrate the scales being considered in different sets of images. Figure adapted under the Creative Commons CC-BY licence from the original article of Jung et al. [24]

Due to the highly individual nature of tissue engineering scaffolds and widely varying scope of research studies, it is not feasible for additive manufacturing software to include the infinite potential parameters that may be desired for print path generation and overall process control. Therefore, some institutions have developed customised in-house software to generate print paths and GCODE, as in the studies of Jung et al. [24], Kang et al. [25], and Ruiz-Cantu et al. [8]. Jung et al. [24] developed an algorithm for generating print paths for heterogeneous cell-laden hydrogel scaffolds with free-form 3D geometry, as outlined in Fig. 3 (a). A key aim of their software was to effectively and intuitively accommodate a range of materials as shown in the print path for a single layer in Fig. 3 (b). Kang et al. [25] also developed custom software to print multiple materials with specifically designed spatial distributions to achieve several 3D tissue structures, and some authors of this review [8] developed custom software to achieve different filament orientations near to the external surface of scaffolds versus the centre to improve control over pore size. As clinical translation continues, it will be important to ensure that software for additive manufacturing systems is suitable for practitioners to use or is able to effectively implement any requirements stated by practitioners.

## Effect of scaffold geometry on performance

**Fig. 4:**
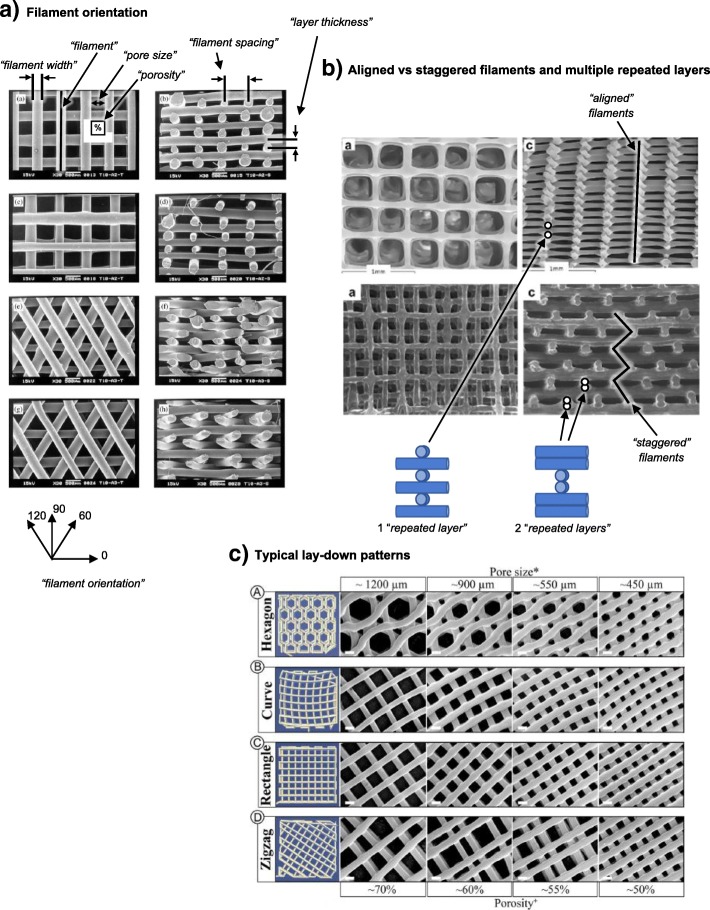
Several printing strategies with different filament positions and orientations: **a** Scaffolds with a 0/90 orientation and 0/60/120 orientation are shown in the top four and bottom four images, respectively [28]. **b** Scaffolds with aligned filaments are shown in the top two images and staggered filaments in the bottom two [29]. **c** Standard infill patterns that are widely used outside the biomedical field but are also used in some tissue engineering scaffold studies. Figures are adapted with permission from the original articles of Zein et al. [28] (Copyright 2001 by Elsevier Science Ltd.), Serra et al. [29] (Copyright 2012 by Acta Materialia, Inc.), and Roohani-Esfahani et al. [6] (Creative Commons CC-BY)

Scaffold geometry influences a variety of factors in tissue engineering. For example, porosity, mechanical properties, and biological performance including cell seeding and cell proliferation may all be affected by the design of the scaffold [7, 26, 27]. The structural geometry of a scaffold is primarily determined by the position and orientation of individual filaments, and a wide range of scaffold geometries have been studied in the literature. Figure 4a shows how different geometries can be achieved by changing the filament orientation on sequential layers. Filaments are frequently oriented at 0° and 90° on alternate layers. This type of structure is herein referred to as a “0/90 orientation”. Alternative scaffold designs utilise a “0/60/120 orientation” [28], in which filament orientations are changed by 60° on each subsequent layer, a “0/45/90/135 orientation” [27], and a “0/72/144/216/288 orientation” [9]. Figure 4b illustrates two other aspects of filament positioning that may be varied [29]: (1) aligned versus staggered filaments, in which filaments are either aligned directly above the similarly oriented filaments on lower layers or staggered in an alternating manner by offsetting their horizontal position, and (2) the concept of “repeated layers”, which refers to several identical layers being printed before the filament orientation is changed. Figure 4c shows the filament orientations that have been widely used as infill for additive manufactured parts both outside and, more recently, within the biomedical field [6].

Many different terms are used in the literature to describe equivalent scaffold features. For example, the extruded filament has been referred to as strut, filament, fibre/fiber, road, rod, raster, extrudate, and other terms. In this review, we give preference to the terms identified in Table 1. These terms are identified in Fig. 4.

**Table 1:** Additive manufacturing terminology used in this review and equivalent terms used in the individual reviewed studies

Terms used in this review	Alternative terms in the literature
Filament	Strut, fibre/fiber, road, rod, raster, extrudate
Filament orientation	Fibre/fiber orientation, lay-down pattern, scan pattern, raster angle, design, print path, layer configuration
Filament spacing	Road gap, filament distance, and similar combinations
Filament width	Strut width, road width, filament diameter, fibre/fiber size, and similar combinations
Layer thickness	Layer height, slice thickness, *z*-increment
Repeated layers	Double layer/triple layer
Staggered filaments (opposed by “aligned filaments”)	Offset layers, diagonal pores
Porosity	Pore fraction, pore volume fraction
Pore size	Pore width

### Effect of scaffold geometry on porosity

The choice of filament position and orientation gives a range of pore shapes, including but not limited to triangular, square, parallelogram, hexagonal, and non-uniform (curved or zigzag path).

Domingos et al. [26] conducted a detailed study of the effect of additive manufacturing process parameters on polycaprolactone (PCL) scaffold porosity. In the study, layer thickness, melt chamber temperature, nozzle travel speed, and extrusion screw speed were varied. The porosity of the fabricated scaffolds ranged from ≈ 49 to 77%, pore size varied from 579 to 711 μm (measured from a top-down view), and pore height varied from 83 to 340 μm (measured from side views of scaffolds). Nozzle travel speed and extrusion screw speed were the most important factors affecting porosity because both of these terms directly affect the size of filaments. Trachtenberg [30] also investigated the effect of process parameters on PCL scaffold porosity but fabricated more dense scaffolds with porosities ranging from ≈ 10 to 60%. In agreement with Domingos et al. [26], they found porosity to be the most affected by parameters that controlled the size of filaments.

**Fig. 5:**
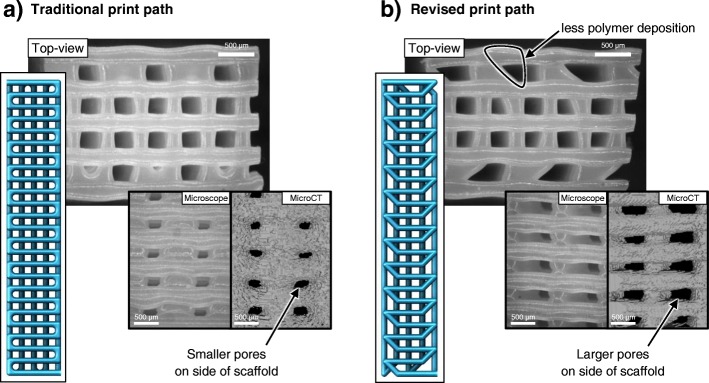
The size of pores on the sides of scaffolds was significantly affected by filament deposition strategy in the study of Ruiz-Cantu et al. For a traditional print path (**a**), the printer nozzle moves to the edge of the scaffold before moving to the start position of the next parallel filament. For the revised print path (**b**), the orientation of the end of filaments was changed by 45°. This prevented excessive polymer deposition at the sides of scaffolds, resulting in larger pores on the external scaffold side surface. Figure adapted with permission from the original article of Ruiz-Cantu et al. [8] (Copyright 2016 by IOP Publishing Ltd.)

Pores on the external surface of scaffolds have received little attention, but in many studies, side views of the scaffolds show pores that are much smaller than internal pores or pores in top-down views of scaffolds [6, 10, 26]. Ruiz-Cantu et al. [8] found that a 5–10-fold increase in porosity at the surface could be achieved, whilst maintaining a similar overall scaffold porosity, by changing the filament lay-down strategy. As shown in Fig. 5, the orientation of each filament was changed by 45° near the edge of the scaffold. This avoided excessive deposition of material at the edge of the scaffold when the extrusion nozzle moved from one parallel filament to the next and therefore increased the size of pores. They also demonstrated that three repeated layers of filaments with the same orientation increased the pore size of surface pores versus one repeated layer.

**Fig. 6:**
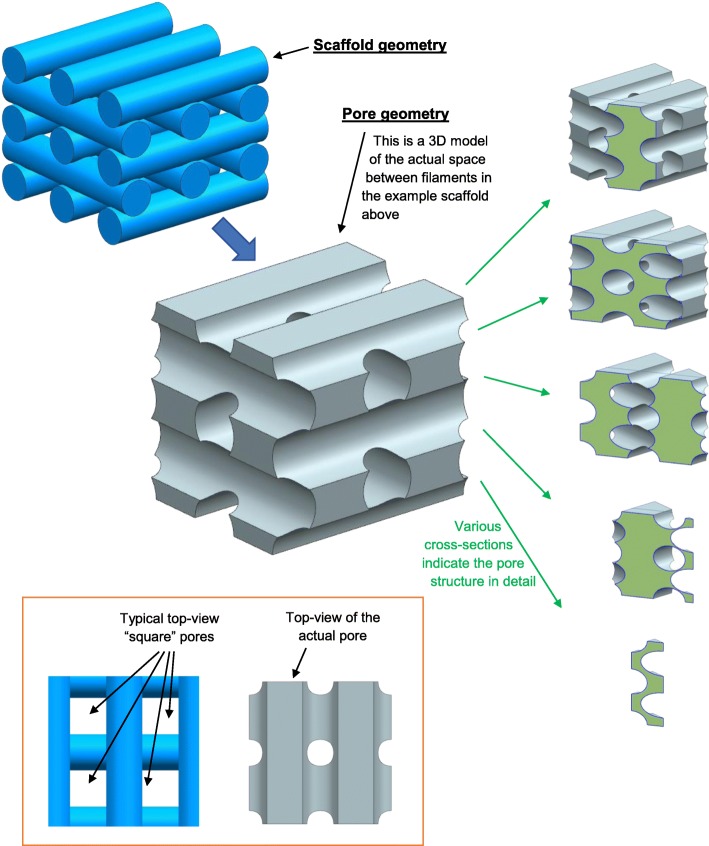
An example scaffold was produced in Siemens NX 11 to demonstrate the complex geometry of pores within tissue engineering scaffolds. A 3D CAD model for an example scaffold is shown in the top left. A 3D CAD model for the volume in between the scaffold filaments (i.e. the “pore”) is shown in the centre of the figure. The five-sectioned images to the right show the cross-sectional geometry of the 3D CAD model for the pore. The two images at the bottom-left show top views of the scaffold and pore to illustrate that “square” pores may be seen in microscopy characterisation of scaffolds, but the actual pore does not have a square morphology

Interconnectivity of pores is a key requirement of tissue engineering scaffolds to ensure mass transfer and oxygen perfusion and can be achieved for a wide range of scaffold designs. Pores are typically shown in images portraying top-down views or side views of scaffolds. This enables pore shape and size to be effectively quantified. However, it may be more appropriate to consider the scaffold to contain a single interconnected pore with filaments running through it, resulting in a highly complex but repeatable pore geometry, as illustrated in Fig. 6. The cross-sectional images on the right-hand side of Fig. 6 illustrate how the pore has a constantly changing complex geometry. The lower images in the figure illustrate how it is an over-simplification to consider pores from a 2D perspective (e.g. considering pores to have a “square” shape), although such a simplification is widely accepted in the literature and can achieve valuable comparisons between scaffolds. For scaffolds with a 0/90 filament orientation, porosity could be considered as a series of long intersecting columnar pores. For a 0/60/120 filament orientation, however, pores may more closely resemble the geometry of a spiral staircase. Such differences between 3D pore shapes are difficult to communicate with static images, and 2D analysis is currently the most accepted approach for scientific reporting. However, recent publications lean towards an increased use of supporting data and the inclusion of 3D models with scientific articles, which may facilitate more complex morphological analysis of pores in the future. This is particularly important for hypotheses or evaluations related to media flow and oxygen or mass transfer through scaffolds.

### Effect of scaffold geometry on mechanical properties

The position and orientation of filaments affect scaffold mechanical properties in a broad and complex manner. For a given scaffold material, porosity may be the most important factor affecting mechanical properties (mechanical integrity reduces with increasing porosity) as demonstrated by several studies in the literature [26, 28, 30–34]. For example, Domingos et al. [26] concluded that “the mechanical performance showed to be highly dependent on the porosity level” for their detailed study of scaffold mechanical properties. Similar findings were made by De Ciurana et al. [34] and Trachtenberg et al. [30], who both developed models to relate mechanical properties to porosity. Too et al. [31, 32], Ang et al. [33], and Zein et al. [28]all also found part strength or elastic modulus to increase as porosity reduced and presented equations to relate mechanical properties to porosity.

Other aspects of scaffold geometry also affect mechanical properties; therefore, this section discusses how mechanical properties are affected by structural geometry even when the porosity is kept constant.

#### Aligned versus staggered filaments

**Fig. 7:**
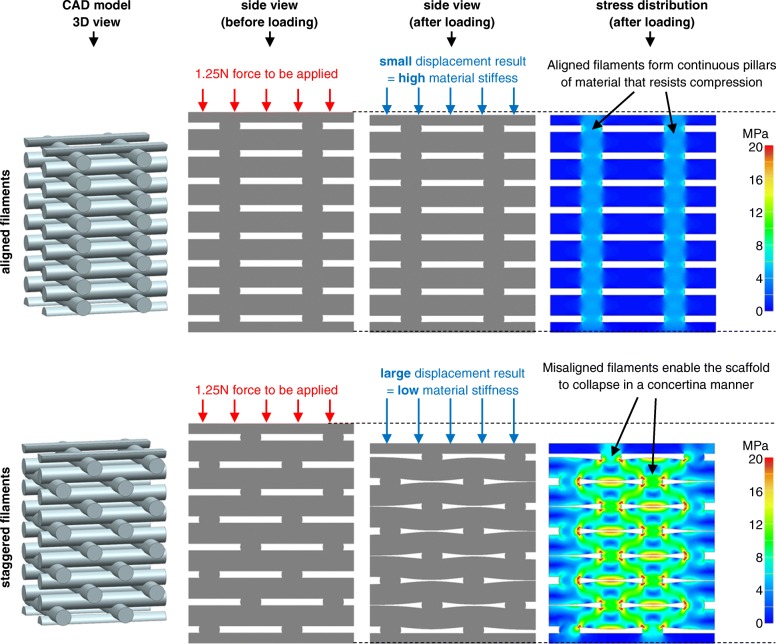
Illustrative FEA example demonstrates the effect of aligned (top row of images) and staggered (bottom row of image) filaments on mechanical stiffness. The four views from left to right indicate the 3D model followed by side views (from left to right): before compression, after compression, and a von Mises stress colour map. The same force is applied to both scaffolds in FEA simulations. In the top scaffold, the filaments are aligned from top-to-bottom and therefore form a continuous pillar of polymer that resists compression. In contrast, the bottom scaffold has staggered filaments and the structure compresses by deformation at hinge points (located at regions of high-stress concentration in the von Mises stress plot). The scaffold collapses in a concertina manner by slightly bending filaments, which results in reduced stiffness versus the top scaffold with a continuous column of polymer

Serra et al. [29] found that scaffolds with staggered filaments had 50–75% lower elastic modulus than scaffolds with aligned filaments (as defined in Fig. 4b). Similarly, Korpela et al. [35] found approximately 40% lower elastic modulus for staggered filaments. Woodfield et al. [11] and Sobral et al. [7] also found similar trends. The illustrative finite element analysis (FEA) examples in Fig. 7 can explain these results based on the mechanisms by which scaffolds collapse. For the aligned filament example (top of Fig. 7), there is a solid column of polymer from top-to-bottom of the scaffold, which exists because filaments all intersect at similar positions. This solid column strongly resists compression. In contrast, for the staggered filament example (bottom of Fig. 7), the structure collapses in a concertina manner; filaments bend slightly, and stress is concentrated at hinge points, as shown in the bottom-right image of von Mises stress distribution.

#### Filament orientation

**Fig. 8:**
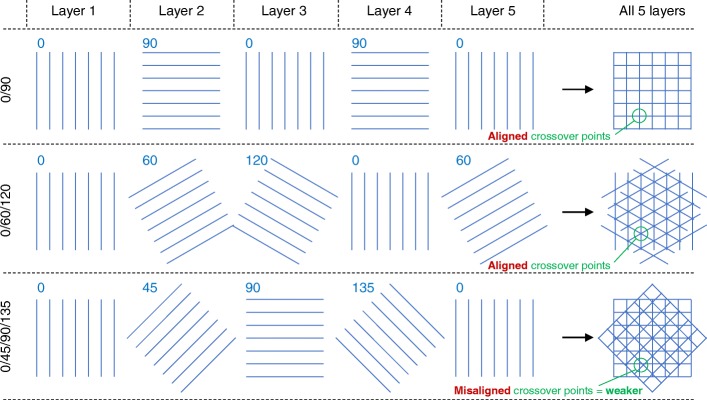
Schematic representation of the first five layers of scaffolds with a 0/90 orientation (top), 0/60/120 orientation (middle), and 0/45/90/135 orientation (bottom). All five layers are overlaid on top of one another in the right-hand column for each of the three different orientations—these represent top-down views of the scaffolds. For 0/90 and 0/60/120 scaffolds, the filaments on different layers all cross through the same points. For the 0/45/90/135 scaffold, filaments on different layers do not pass through the same points, which may result in a weaker structure

Moroni et al. [36], Tellis et al. [37] and Domingos et al. [27] found polymer scaffolds with a 0/45/90/135 orientation to have a lower elastic modulus than samples with a 0/90 or 0/60/120 orientation. This difference can be explained by the alignment of filaments on subsequent layers, as illustrated in Fig. 8, which shows the first five layers of scaffolds with 0/90, 0/60/120, and 0/45/90/135 orientations. On the right-hand side of the figure, the five layers are all overlaid. For 0/90 or 0/60/120 orientations (top two rows in Fig. 8), the filaments cross over at the same positions on every layer. This generates a solid column of polymer from top-to-bottom of the scaffold and results in a high elastic modulus, as discussed in relation to Fig. 7. In contrast, 0/45/90/135 scaffold filaments cross over at different positions on each layer (bottom right of Fig. 8) due to fundamental trigonometric principles. Therefore, elastic modulus may be lower, in agreement with the experimental findings [27, 36, 37]. Similarly, cross-over points are staggered for a 0/72/144/216/288 orientation, which may therefore have a lower modulus than a 0/60/120 orientation, as found experimentally by Hutmacher et al. [9]. Other studies [34, 38] have considered several filament orientations, but porosity varied between scaffold designs so it is not possible to directly quantify the effect of filament orientation.

#### Repeated layers

Moroni et al. [36] found scaffolds with two repeated layers to have a lower elastic modulus than those with alternating layers. They hypothesised that longer pores along the compression axis lead to a mechanically weaker structure. In contrast, Ruiz-Cantu et al. [8] found 0/90 scaffolds with one, two, or three repeated layers to all have similar elastic moduli. Serra et al. [29] also compared scaffolds with one and two repeated layers, but direct comparison is difficult because they also varied the alignment of filaments. Varying the number of the repeated layer has a large impact on pore size [8], but more studies comparing the repeating layers are required for the influence on mechanical properties to be understood.

#### Other lay-down patterns

Roohani-Esfahani et al. [6] studied several filament lay-down patterns for tissue engineering scaffolds fabricated from a bioactive glass-ceramic consisting of strontium-doped hardystonite and gahnite. They utilised hexagonal, curved, rectangular, and zigzag patterns with pore sizes ranging from 450 to 1200 μm and achieved scaffolds with similar strengths to the cortical bone for overall scaffold porosities of approximately 50 to 65%. The hexagonal lay-down pattern resulted in a greater compressive strength, flexural strength, and fatigue life than other designs because it facilitated a higher contact area between additive manufactured layers. This supports the earlier discussion in relation to Figs. 7 and 8 because a greater contact area between layers generates a wider solid column of polymer from top-to-bottom of the scaffold. Shao et al. [10] also found a hexagonal lay-down pattern to have a greatest strength and elastic modulus for scaffolds fabricated from calcium silicate and bioactive glass.

#### Anisotropic properties

Zein et al. [28] demonstrated that scaffold mechanical properties depend on the loading direction. In their study, PCL scaffolds with a 0/90 orientation had lower strength and elastic modulus when loaded in the build direction (normal to the print bed). This finding was more pronounced for a 0/90 orientation versus 0/60/120, which demonstrates that filament orientation affects anisotropic mechanical properties. As the clinical readiness of scaffolds increases, studies will need to include more analysis of anisotropic properties to ensure that the scaffolds are being effectively characterised and reduce the risk of unanticipated weaknesses in particular loading directions. Currently, most studies focus on mechanical properties in the build direction.

#### Degradation of mechanical properties

The effect of scaffold geometry on degradation and resorption have not been extensively researched, although there have been some in vitro degradation studies [10, 39]. For example, Shao et al. [10] found that calcium silicate and bioactive glass scaffolds demonstrated greater strength increases for a 0/90 filament orientation versus a hexagonal lay-down pattern. Domingos et al. [39] found the degradation rate of PCL scaffolds to be affected by porosity and pore size, whilst 0/90, 0/60/120, and 0/45/90/135 filament orientations were all found to have similar degradation rates.

#### Significance in relation to mechanical properties of biological tissue

In many clinical applications, it is desired for scaffolds to have similar mechanical properties to the natural biological material. This is especially true for tissues that must withstand bending, torsion, and pressure, such as the skin, cartilage, and load-bearing bone. The stiffness and strength of a scaffold are two distinct properties: stiffness (also referred to as elastic modulus) is defined by how much a material deforms in response to a given force, whereas strength refers to the maximum force that can be sustained before the material fails.

The elastic modulus of biological tissues ranges greatly but is in the regions of < 1 MPa for soft tissues, 0.3 to 20 MPa for cartilage [40, 41], 100 to 500 MPa for cancellous bone [42], and 12,000 to 20,000 MPa for cortical bone [42, 43]. The elastic modulus for engineered tissues varies greatly in the literature: from less than 1 MPa to over 1000 MPa depending on the material: hydrogel scaffolds, especially natural hydrogels, may have low elastic modulus < 1 MPa; polymeric scaffolds may have elastic moduli in the range of 3 to 12 MPa for polybutylene terephthalate [37], 10 to 160 MPa for polycaprolactone [8, 26], or 200 to 1200 MPa for polylactide [34]; ceramic scaffolds can also achieve high elastic moduli in the region of 150 to 800 MPa or greater [10, 44]. Whilst selecting an appropriate material for replaced tissue is necessary, it is also critical that the position and orientation of filaments are tailored to achieve the desired porosity and mechanical properties. In some cases, it may be desirable to reduce the stiffness of a scaffold to better-match biological tissue stiffness.

Scaffold strength is also critically important and may be in the range of 14 to 59 MPa for cartilage [41], 1 to 12 MPa for cancellous bone [42], and 50 to 190 MPa for cortical bone [42]. Tissue engineering scaffold strengths may range from 1 to 9 MPa for polycaprolactone [9, 26, 27, 45], 60 to 130 MPa for ceramic polymer composites [46], and 16 to 180 MPa for ceramic [6, 10, 44]. As with scaffold stiffness, choosing an appropriate material is essential to achieve an appropriate strength, but it is also critical to effectively design the scaffold geometry since this can affect strength by almost an order of magnitude [26].

### Effect of scaffold geometry on cell seeding and proliferation

Scaffold pore size affects cell seeding and cell proliferation [47–50]. Temple et al. [51] demonstrated this for human adipose-derived stem cells by adjusting the filament spacing in PCL scaffolds. They varied pore size from approximately 200 to 2000 μm and found that the most uniform cell seeding was achieved in scaffolds with ≈ 800 μm pores. In contrast, Lee et al. [12] found that a pore size of 500 μm was too large for pre-osteoblasts in poly (propylene fumarate) scaffolds and that 350 μm was the ideal size for proliferation. For human mesenchymal stem cells seeded on PCL scaffolds with a 0/90 orientation, Domingos et al. [27] found greater cell proliferation for larger pores within the range of 245 to 433 μm. Similarly, Park et al. [52] found that chondrocytes more easily penetrated PCL scaffolds with larger pores in the range of 100 to 300 μm. These results suggest that the size of pores must be optimised for specific cell types and culture conditions.

Whilst many studies investigate pore size inside the scaffold, some authors of this review investigated the effect of pores on the external surface of the scaffold [8], which are often smaller than internal pores. For chondrocytes on PCL scaffolds, it was shown that increasing the porosity at the surface of the scaffold enables up to a 55% increase in dynamic cell seeding efficiency and up to 110% greater cell proliferation over 14 days.

Even if the pore size is kept constant, Sobral et al. [7] found that scaffold geometry greatly affects cell seeding efficiency for osteosarcoma cells on scaffolds fabricated from a cornstarch polycaprolactone blend. The surface area of the scaffold was found to be important along with the configuration of filaments and its impact on flow conditions of the cell suspension during dynamic seeding. In scaffolds with aligned filaments, in which pores ran directly from top to bottom of the scaffold, the cell suspension may have travelled through relatively quickly and thus not facilitated effective cell attachment. Cell seeding efficiency increased in scaffolds with staggered filaments, potentially because the cell suspension had to continuously change direction as it passed through the scaffold. The authors hypothesised that this led to a slow flow rate and increased opportunity for cell attachment. Their results show that even when many aspects of the scaffold design are kept constant, the orientation and layout of filaments are critically important for biological performance. Similarly, Lee et al. [12] found improved cell proliferation for pre-osteoblasts in poly (propylene fumarate) scaffolds with staggered filaments versus aligned filaments. Although microstereolithography was used as the fabrication process by Lee et al. [12], it has been discussed here because the scaffold geometries were similar to the material extrusion additive manufactured scaffolds. In contrast, Korpela et al. [35] seeded fibroblasts onto PCL scaffolds and found no significant difference between 0/90 scaffolds with aligned or staggered filaments. They also found no significant difference between 0/90 scaffolds and 0/60/120 scaffolds, in contrast to Domingos et al. [27] who found greater proliferation of human mesenchymal stem cells in PCL scaffolds with a 0/90 orientation versus 0/60/120 and 0/45/90/135 orientations. Hutmacher et al. [9] found osteoblast-like cells to have a higher proliferation rate in the first 2 weeks on PCL scaffolds with a 0/60/120 orientation versus 0/72/144/216/288 orientation, but slower for weeks 3–4. Laronda et al. [53] varied filament orientation in gelatin scaffolds to optimise pore geometries for murine follicle survival and function. They found follicles to demonstrate a better survival rate when they contacted multiple filaments (i.e. residing in the corner between filaments). Follicles contacted a greater number of filaments on average in scaffolds with 0/30/60/90/120 or 0/60/120 orientation versus a 0/90 orientation, and therefore, these scaffold designs achieved an improved survival rate. From these data, it can be concluded that the effect of filament orientation on cell proliferation varies considerably in the literature. This is likely due to the highly complex nature of cell culture.

From the above-mentioned studies, it is clear that the position and orientation of filaments greatly affect cell seeding and proliferation. However, because study outcomes vary greatly, further research is required to understand how the fundamental mechanisms of cell attachment and proliferation are affected by scaffold geometry. In addition, longer-term studies investigating tissue formation, immune response, and scaffold resorption for multiple scaffold geometries are required. The reader is directed to the review of Murphy and Atala for a more comprehensive evaluation of bioprinting tissues [54].

## Innovative strategies for polymer deposition

The previous section reviewed studies that directly compared scaffold geometries achieve through different strategies for filament placement and orientation. Most of the studies used standard additive manufacturing software to vary the filament deposition strategy. This section reviews studies that have reported innovative strategies for polymer deposition to achieve novel structures and geometries.

### Curved layers

**Fig. 9:**
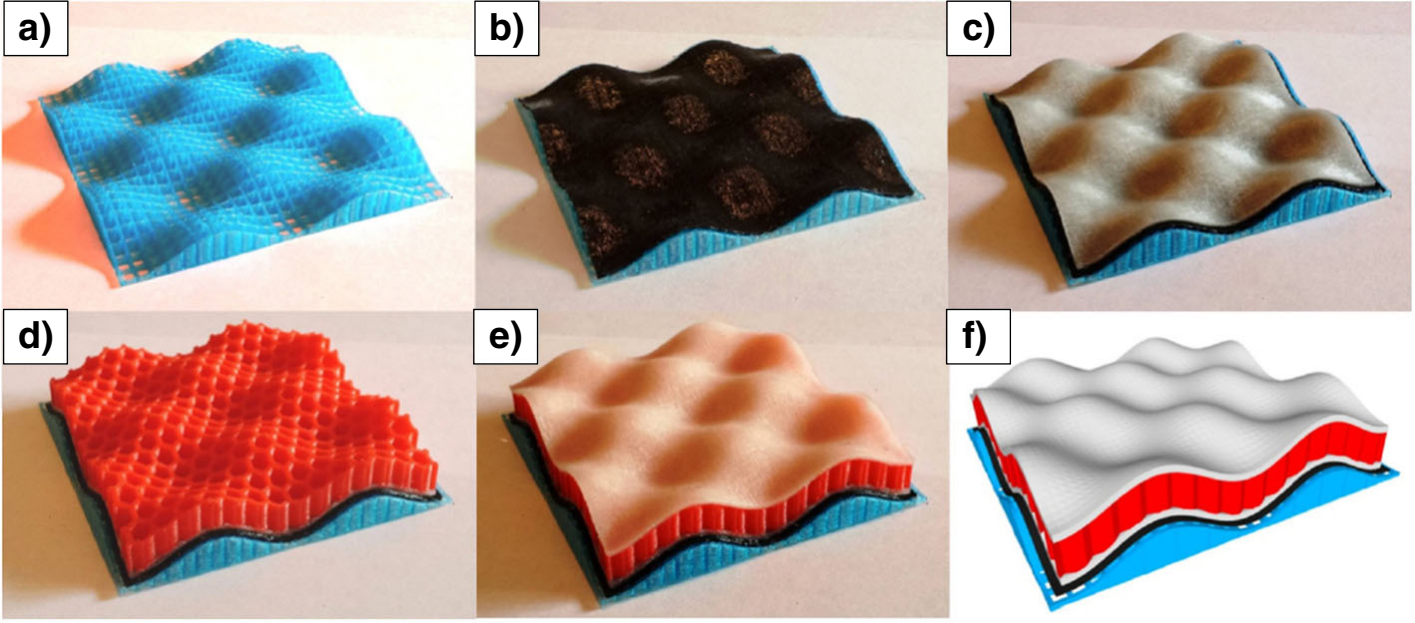
Curved layers were used in order to print a multi-material object with the following stages: **a** support material, **b** sacrificial layer, **c** lower skin, **d** porous core, and **e** upper skin. **f** CAD model of the object. Figure adapted under the Creative Commons CC-BY licence from the original article of Allen and Trask [55]

Most additive manufactured parts are produced layer-by-layer, whereby all filaments are extruded for a single layer (with the nozzle at a constant height above the print bed) before the nozzle moves up by the “layer thickness” to begin printing the next layer. Allen and Trask [55] demonstrated an alternative strategy in which “curved layers” were printed by moving the nozzle away/closer to the print bed during the deposition of a single filament. In their study, four different materials were used: first, a support structure was printed, then a sacrificial dissolvable layer, then lower skin, then core, and finally top skin, as shown in Fig. 9. Due to the curved shape, a traditional layer-by-layer strategy for polymer deposition would have required all four materials to be printed on every layer; this would have resulted in hundreds of material changes (and an increased printing time), as opposed to just four changes for the entire print. The main disadvantage of curved layers is that generation of the print-toolpath is more complex. For anatomically shaped scaffolds, curved layers can potentially enable improved pore consistency and mechanical properties [56].

### Variable layer thickness

**Fig. 10:**
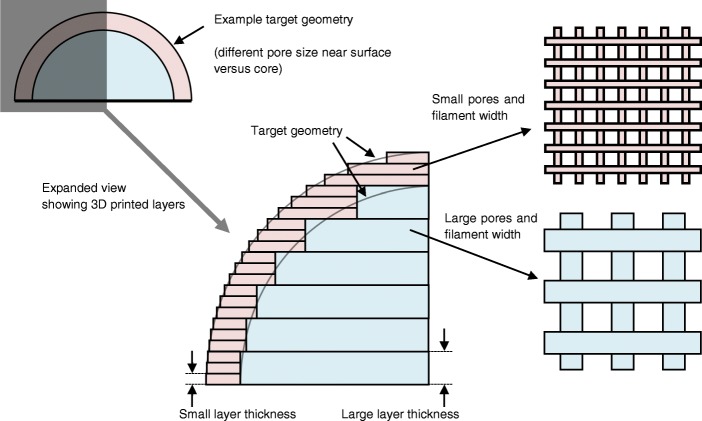
Illustrative example of varying the layer thickness at different positions within a part to achieve different scaffold geometries near the surface and in the centre of a scaffold

Many studies were published before the year 2000 for adaptive slicing algorithms in which the layer thickness varied during fabrication [57–59]. In most cases, the proposed benefits were to improve geometric accuracy and reduce fabrication time. This concept has received less attention recently but could be utilised for scaffold fabrication to achieve different filament and pore geometries at different positions within a scaffold. Figure 10 illustrates how adaptive layer thickness could enable different pore sizes at the surface versus inside a scaffold. Whilst variation of layer thickness directly affects pore size when viewing scaffolds from the side, Khoda et al. [60] demonstrated a print path planning algorithm to generate variational and more accurate pore sizes when viewing scaffolds top-down.

### Adjustment of nozzle height and extrusion rate

**Fig. 11:**
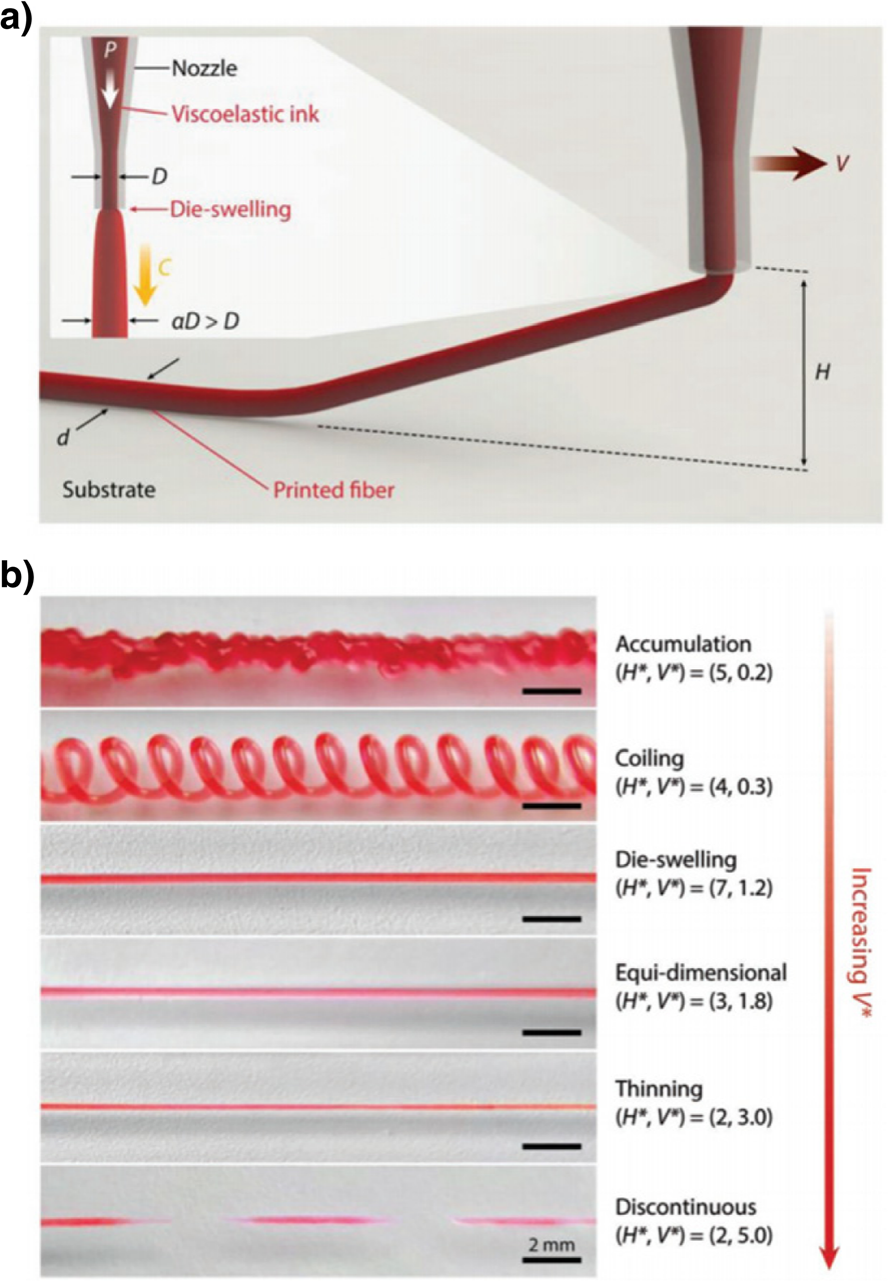
A new strategy for material extrusion additive manufacturing in which the speed and height of the print nozzle were changed to achieve accumulation or thinning of extruded silicone hydrogel. **a** The concept of raising the nozzle above the print bed and controlling the extrusion rate and nozzle speed. **b** The resulting printing characteristics achieved with various settings for non-dimensionalised parameters for print height (H*) and nozzle speed (V*). Figure reproduced with permission from the original article of Yuk and Zhao [62] (Copyright 2017 by WILEY-VCH Verlag GmbH & Co. KGaA, Weinheim)

Two publications in the last 12 months have investigated a new strategy for material extrusion additive manufacturing, in which the speed of the nozzle and the rate of material extrusion were adjusted to achieve new structures [61, 62]. Figure 11 shows the strategy used by Yuk and Zhao [62]: by setting a low extrusion rate relative to nozzle speed, the filament was stretched to be narrower than the nozzle diameter, whereas setting a high extrusion rate caused the filament to either widen or fold on itself (“coiling” or “accumulation” in Fig. 11) depending on how far the nozzle was from the print bed. The authors suggested that innovations and applications of material extrusion additive manufacturing have been severely hampered by the limitations of typical deposition strategies. Takahashi and Miyashita [61] also undertook a detailed study varying the extrusion rate and height of the nozzle but for polylactide as opposed to a silicone hydrogel. They demonstrated capabilities to print a range of structural geometries with a focus on achieving different surface textures.

## Future outlook

It may be argued that research into additive manufactured tissue engineering scaffolds has been both driven and limited by the capabilities of additive manufacturing systems: on one hand, the introduction of additive manufacturing to the tissue engineering field enabled ground-breaking scaffold geometries to be manufactured; on the other hand, almost all studies have produced relatively similar scaffolds by selecting a filament placement strategy from the options provided in additive manufacturing software. The user-friendly attributes of additive manufacturing software have enabled significant research over the last decade. However, the true capabilities of additive manufacturing systems are being under-utilised because it is unfeasible for generic software supplied by system manufacturers to fully address the wide-ranging needs of all researchers (the number of software parameters would become impractical). Therefore, in a few cases, custom software has been developed to offer more precise control over specific aspects of the additive manufacturing nozzle position and print path—for example, to enable precise placement of different cell-laden hydrogels in tissue constructs [25]—but this is uncommon, and the custom software typically shares many similarities with off-the-shelf software. Fundamentally, a material extrusion additive manufacturing system is simply a robot that can place material in a chosen position, and there is a wide scope for entirely new and novel structures to be produced [61, 62]. For example, by carefully designing the arrangement of filaments, auxetic scaffolds could be fabricated [63], which would provide extremely different mechanical stress conditions for cells (versus regular scaffolds) and replicate the auxetic properties of some natural tissues including the skin, bone, and endothelium tissue [64]. In addition to experimental developments, computational models should also be developed, for example, to simulate the geometry and properties of tissue engineering scaffolds [65]. Engineers and medical biological researchers must continue to collaborate, and custom software should be shared if possible (e.g. as supplementary information with journal publications).

New additive manufacturing capabilities (machine hardware and software) can be readily developed, but this requires further research to understand the relationship between scaffold geometry and the complex biological requirements of tissue regeneration. This research will inform engineers of ideal scaffold geometries and direct future engineering developments in additive manufacturing. Even though knowledge of the complex extracellular matrix, in terms of design and geometry, of many tissues and organs currently exists, the requirements to recreate this complex matrix are not fully understood. For example, the overall structure and design of ear cartilage extracellular matrix is known: the tissue consists of a dense collagen, elastin, and glycosaminoglycans matrix, with chondrocytes embedded in lacunae. However, the effect of the structure of this matrix on cellular behaviour is not yet known. Therefore, designing a scaffold structure that recapitulates the influence of this matrix cannot be achieved at present. Some advanced printing capabilities have been developed, such as gradient structures, but through engineering collaboration, there is huge potential to use additive manufacturing systems in more advanced and more customised ways. In many cases, this only requires adapted additive manufacturing software, not hardware modification, such as non-constant filament width, variable layer thickness, curved layers, and highly controlled filament orientations. These, and many other, parameters are currently not being controlled in tissue engineering scaffolds—likely because other parameters that can be more easily varied are still not fully understood in terms of their impact on biological performance. It is also important to relate these parameters to the requirements of the implant, as requested by practitioners on an individual-patient basis.

Whilst several studies have considered the effect of filament position and orientation on cell seeding and proliferation, few consider longer-term performance, and therefore, optimisation of the scaffold design for tissue formation is not yet possible. The lack of such studies is probably due to the complexity and expense of long-term in vitro and in vivo studies. By contrast, mechanical characterisation can be completed relatively quickly with low costs; hence, the large number of studies are reviewed above in the “Effect of scaffold geometry on mechanical properties” section, although the analysis of anisoptropic mechanical properties (not widely studied) will become more critical going forwards because multi-directional stresses must be sustained in many clinical applications. The immune response to different print paths has not been studied as far as the authors are aware, although it is feasible for additive manufacturing to control certain factors that influence bacterial attachment and biofilm formation, as well as modulate localised immune responses. For example, optimising scaffold architectural parameters can prevent bacterial colonisation and reduce the risk of implant rejection due to infection or inflammation [66]. Similarly, degradation and resorption of scaffolds have not been extensively researched and require further investigation, particularly in combination with tissue formation and maturation. As cell seeding and proliferation studies develop more comprehensive understandings of the effect of geometry on biological requirements and responses, there will be greater justification to undertake long-term in vitro or in vivo studies. Since few long-term studies directly compare multiple printing strategies or print paths, it is important that systematic reviews and meta-analyses are undertaken to draw confident understanding across multiple studies.

## Conclusion

This paper identified current research into the effect of additive manufactured scaffold geometry on porosity, mechanical properties, and biological performance. It focused on the position and orientation of filaments, also referred to as the lay-down pattern or print path. Analysis of multiple comparable studies enabled new understanding of how mechanical properties are affected by the alignment of filaments across multiple additive manufactured layers. No general consensus was found regarding the optimal scaffold geometries for biological performance, indicating a need for further research with a medical biology focus. Current research studies have considered a relatively narrow range of scaffold geometries: typically, filaments of a constant size are arranged in a regular repeating manner over sequential layers. This is because most bioprinting software packages have similar capabilities. There is great potential for new advanced polymer-deposition strategies, with custom additive manufacturing software, to create novel scaffold structures that more effectively mimic the native tissue environment.
